# *Angiostrongylus cantonensis* Nematode Invasion Pathway, Mallorca, Spain

**DOI:** 10.3201/eid2806.212344

**Published:** 2022-06

**Authors:** Sofia Delgado-Serra, Jessica Sola, Nieves Negre, Claudia Paredes-Esquivel

**Affiliations:** University of the Balearic Islands, Palma, Spain (S. Delgado-Serra; C. Paredes-Esquivel);; Consorci per a la Recuperació de la Fauna de les Illes Balears, Santa Eugènia, Spain (J. Sola, N. Negre)

**Keywords:** Angiostrongylus cantonensis, infection, angiostrongyliasis, human eosinophilic meningitis, meningitis/encephalitis, rat lungworm infection, helminths, parasites, sentinel species, animal diseases, central nervous system, parasitic infections, nematodes, invasion pathway, vector-borne infections, hedgehogs, zoonoses, Mallorca, Spain

## Abstract

Neural angiostrongyliasis is an emerging zoonosis caused by the rat lungworm, *Angiostrongylus cantonensis*. In humans, infection with this nematode often results in eosinophilic meningitis and other severe disorders of the central nervous system. Europe was deemed a nonendemic region until 2018, when *A. cantonensis* worms were detected on the Mediterranean island of Mallorca, Spain, a tourism hotspot. Since that time, a sentinel surveillance system and a molecular approach have been used to follow the invasion path of the rat lungworm on the island. *A. cantonensis* worms have been found in animals from 8 locations on the island over 3 consecutive years. Our preliminary results show a recognizable pattern of clinical signs in infected hedgehogs and a single mitochondrial haplotype circulating in Mallorca. We present strong evidence confirming that the rat lungworm has successfully established and colonized an island in Europe and discuss observations and possible strategies for its early detection across continental Europe.

The rat lungworm, *Angiostrongylus cantonensis*, infects animals and humans. Although this nematode species is recognized as the main etiologic agent of eosinophilic meningitis (*1*), infection might result in other central nervous system disorders (*2*). Clinical manifestations are aggravated by movement and subsequent death of the worms in the central nervous system, causing physical lesions and inflammation in accidental hosts (*3*). In humans, severe headache, neck stiffness, paresthesia, convulsions, urinary failure, visual impairment, and other symptoms, occasionally leading to coma and death, have been reported (*1*,*4*).

The life cycle of *A. cantonensis* worms includes rats as definitive and gastropods as intermediate hosts; crustaceans, planarians, amphibians, reptiles, and fish might act as paratenic hosts (*2*). More than 20 vertebrate species, including humans, have been reported as *A. cantonensis* lungworm accidental hosts (*5*). This long list of vertebrate hosts includes nonhuman primates (*6*), marsupials (*7*), bats (*8*), horses (*9*), dogs (*10*), birds (*11*), and more recently, hedgehogs (*12*). The role of hedgehogs in the transmission of this parasite remains to be clarified.

*A. cantonensis* worms were detected in Canton, China, infecting the lungs of rats (*13*) and a decade later, in the cerebrospinal fluid of a person from Taiwan (*14*). For decades, disease-endemic areas were limited to the Pacific basin and Southeast Asia, but this parasite has spread to new territories at an alarming rate (*1*). The invasion of *A. cantonensis* lungworms has been associated with unintended importation of infected rats and gastropods on ships (*2*,*15*). Almost 3,000 cases of human neuroangiostrongyliasis have been reported (*16*) from 30 territories (*3*), although the prevalence might be higher (*17*).

Europe was considered to be nonendemic for *A. cantonensis* worms until 2018 when the parasite was reported infecting the brains of 2 hedgehogs on the Mediterranean island of Mallorca (*12*). Although the rat lungworm had been previously reported on Tenerife, a subtropical, non-European overseas oceanic island (*18*), its detection in Mallorca is an indisputable indication of its presence in Europe (*5*). Mallorca is a major Mediterranean tourism hotspot, highly interconnected with continental Europe. After the detection, the question remained whether *A. cantonensis* nematodes could survive the temperate winters of Europe. The purpose of this study was to use sentinel surveillance for symptomatic fauna to confirm whether the rat lungworm has been successfully established on Mallorca.

## Methods

### Surveillance Strategy

We conducted sentinel surveillance of hedgehogs that had signs of disease during 2018‒2020 for early detection of *A. cantonensis* lungworm‒positive animals on Mallorca. Availability of animals was contingent on local citizens providing injured, ill, or orphaned North African hedgehogs (*Atelerix algirus*) to the Consorci per a la Recuperació de la Fauna de les Illes Balears wildlife hospital. Animals showing neurologic clinical signs were hospitalized, and their behavior was observed daily.

When possible, a blood sample was obtained from the animal’s jugular vein and sent to an external laboratory (Laboratorio Echevarne S.A., https://laboratorioechevarne.com) for hematologic and clinical chemistry analyses. Blood extraction was not always possible in severely ill or dehydrated hedgehogs. We euthanized critically ill animals to avoid suffering and then subjected them to necropsy, performed in a BioSafety Level 2 facility, according to the regulations of the University of the Balearic Islands. We kept lungs, heart, and head frozen for further analysis.

### Detection and Morphologic Identification

We opened preserved skulls by using a scalpel and making 2 parallel incisions along the frontal and parietal bones to access the brain underneath. We completely removed the brain and macroscopically examined the interior of the skull and the subarachnoid space of the brain by using a stereomicroscope (magnification ×10–40). We conducted external examination of the lungs, heart and pulmonary arteries according to the same procedure. We collected nematodes from the brain and the skull’s inner surface.

During 2018, we detected parasites macroscopically, During 2019 and 2020, we changed the method approach and used a tissue digestion technique after the visual inspection. When worms were present, we tentatively identified them as *A. cantonenisis* nematodes by their typical barber’s pole appearance, which results from spiral disposition of the blood-filled intestine and the white uterine tubes in fully developed female worms. This characteristic can be observed in other *Angiostrongylus* species. Using the morphologic keys of Chen (*13*) and Kinsella (*19*), we also identified male nematodes on the basis of characteristics of the copulatory bursa, with a small dorsal ray, shorter than the externodorsal ones, and by the presence of long spicules (1–1.4 μm). We identified female worms on the basis of the form of their ventrally curved posterior end. We distinguished adults from larvae by their body size and development of the sexual apparatus.

### Molecular Identification and Phylogenetic Analysis

We conducted molecular analysis to confirm the morphologic identifications. We extracted genomic DNA by using an NZY Tissue gDNA Isolation Kit (Nzytech, https://www.nzytech.com) and amplified a fragment of the cytochrome c oxidase subunit I (COI) gene region by PCR using primers COI forward, 5′-TTTTTTGGGCATCCTGAGGTTTAT-3′, and COI reverse, 5′-TAAAGAAAGAACATAATGAAAATG-3′ (*20*). The 50-μL PCR contained 2 μL of genomic DNA, 2 μL of each primer (10 mmol/L), 2 μL of 50 mmol/L MgCl_2_, 25 μL of Taq Master Mix (Supreme NZY*Taq*II 2x Green Master Mix; Nzytech), and 17 μL of water. We performed PCRs in a Verity Thermo Cycler (Applied Biosystems, https://www.thermofisher.com) as follows: 1 cycle of initial denaturation at 95°C for 3 min; followed by 35 cycles at 95°C for 30 s, 50°C for 30 s, and 72°C for 1 min; and a final extension at 72°C for 10 min.

We visualized PCR products by electrophoresis on a 2% agarose gel containing Pronasafe Nucleic Acid Stain (Conda Laboratories, https://www.condalab.com). We purified samples by using an NZYGelpure Purification Kit (Nzytech) according to manufacturer specifications. We performed Sanger sequencing by Sistemas Genómicos S.L. (https://www.sistemasgenomicos.com). One *A. cantonensis* specimen/infected hedgehog was sequenced.

We conducted BLAST analysis (https://blast.ncbi.nlm.nih.gov) of the resulting sequences and used the GenBank database to confirm the identification of the parasites. We retrieved the top 78 hits corresponding with COI sequences of *A. cantonensis* nematodes for further phylogenetic analysis. We aligned retrieved sequences from GenBank and those obtained in this study by using CodonCode Aligner version 9.0.1 (CodonCode Co., https://www.codoncode.com). We inferred a maximum-likelihood phylogenetic tree by using MEGAX software (https://www.megasoftware.net) with Kimura 2-parameter and 500 bootstrap replicates.

## Results

In a 3-year period, 8 animals that had signs of disease were rescued by local citizens from different parts of Mallorca. These animals had clinical signs compatible with a neurologic disease: astasia, pelvic limb ataxia, atonia, asthenia, paresis, and behavioral decay. Five of these animals were females (3 adults, 2 juveniles) and 3 were males (2 adults, 1 juvenile). The age of the hedgehogs was calculated according to Garcia-Salguero et al. (*21*). The first 2 hedgehogs received were reported previously (*12*). The common clinical signs in infected hedgehogs were astasia, defined as the inability to stand and walk; lateral recumbency (present in all examined hedgehogs), defined as lying on their side; and bicycling movement (present in 6/8 hedgehogs), defined as a consistent, synchronized movement of the limbs (Video). Bicycling often resulted in skin lacerations.

**Video:** Recognizable pattern of clinical signs in hedgehog infected with *Angiostrongylus cantonensis* lungworm, Mallorca, Spain, showing typical clinical manifestations: astasia, lateral recumbency, and bicycling movement of the limbs.

**Figure 1:**
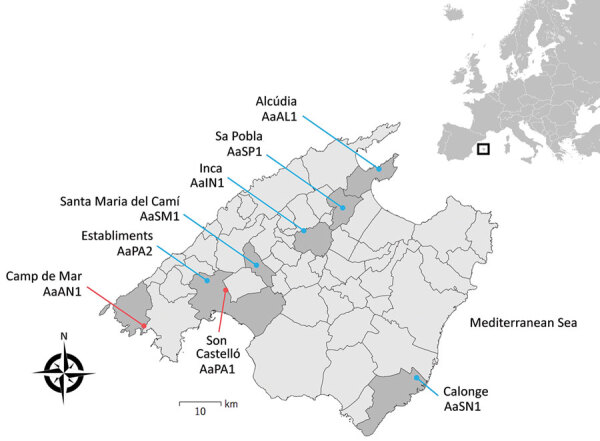
Geographic location of hedgehogs infected by *Angiostrongylus cantonensis* rat lungworms, Mallorca, Spain, 2018–2020. Blue indicates new cases reported in this study (see Table for details), and red indicates cases reported previously (*12*). Inset shows location of Mallorca off the coast of southwestern Europe.

Infected hedgehogs were found in 8 localities from 7 of municipalities in Mallorca (Table; Figure 1). These locations varied from typical coastal places (hedgehogs AaAL1 and AaAN1) (the abbreviation Aa indicates the name of the hedgehog species [A. algirus]), in which tourism is the most prominent economic activity, to traditional inland rural areas dedicated to farmland (hedgehogs AaSP1 and AaSM1) (Figure 1). With the exception of hedgehog AaSN1, all specimens were found in municipalities located at the foot of the eastern foothills of the Tramuntana Mountain range. Two hedgehogs showed positive results during 2018 and 2019, and 4 hedgehogs showed positive results during 2020. All positive hedgehogs harbored *A. cantonensis* adults. None of the female worms had eggs.

**Table:** Details and clinical information for *Angiostrongylus cantonensis* lungworm‒infected hedgehogs hospitalized at the Consorci per a la Recuperació de la Fauna de les Illes Balears wildlife hospital, Mallorca, Spain

Hedgehog specimen	Location	Date	Clinical manifestations	No. lungworms recovered		Helminth co-infections
				On skull	In brain	
AaAN1*	Camp de Mar (Andratx)	2018 Oct 13	Pelvic limb ataxia, atonia, asthenia, behavioral decay, lateral recumbency	0	1	None
AaPA1*	Son Castelló (Palma)	2018 Oct 23	Pelvic limb ataxia, atonia, behavioral decay, lateral recumbency	0	5	None
AaSP1	Sa Pobla	2019 Nov 11	Asthenia, astasia, bicycling movements, lateral recumbency, skin lacerations	1 male	0	*Crenosoma striatum* (lungs)
AaAL1	Alcúdia	2019 Dec 23	Astasia, bicycling movements, lateral recumbency	0	2 female, 2 male, 4 damaged specimens	*Crenosoma striatum* (lungs)
AaSM1	Santa Maria del Camí	2020 Jan 28	Astasia, bicycling movements, lateral recumbency	0	11 female, 191 male, 7 damaged specimens	None
AaIN1	Inca	2020 Oct 28	Astasia, bicycling movements, skin lacerations, lateral recumbency	0	6 female, 3 male, 9 damaged specimens	*Crenosoma striatum* (lungs)
AaPA2	Establiments (Palma)	2020 Nov 26	Astasia, bicycling movements, lateral recumbency	2 female, 1 male, 1 damaged specimen	33 female, 20 male, 11 damaged specimens	*Crenosoma striatum* (lungs)
AaSN1	Calonge (Santanyí)	2020 Dec 28	Astasia, repetitive cycling movements, lateral recumbency	0	2 female	*Crenosoma striatum* (lungs)

*Infected hedgehogs previously reported (*12*).

Infected hedgehogs were found during autumn to early winter, specifically during September, October, and December (Table). None of the *A. cantonensis* specimens were found in the lungs or hearts of infected hedgehogs. All but 2 positive hedgehogs were co-infected with the lungworm *Crenosoma striatum*. Hematologic analysis could only be conducted for 2 infected hedgehogs. Hedgehog AaSP1 had a blood eosinophil count of 4% and an absolute blood count of 0.836 × 10^3^ cells/μL, and hedgehog AaAL1 had a blood eosinophil count of 2% and an absolute blood count of 0.208 × 10^3^ cells/μL.

### DNA Assessment

**Figure 2:**
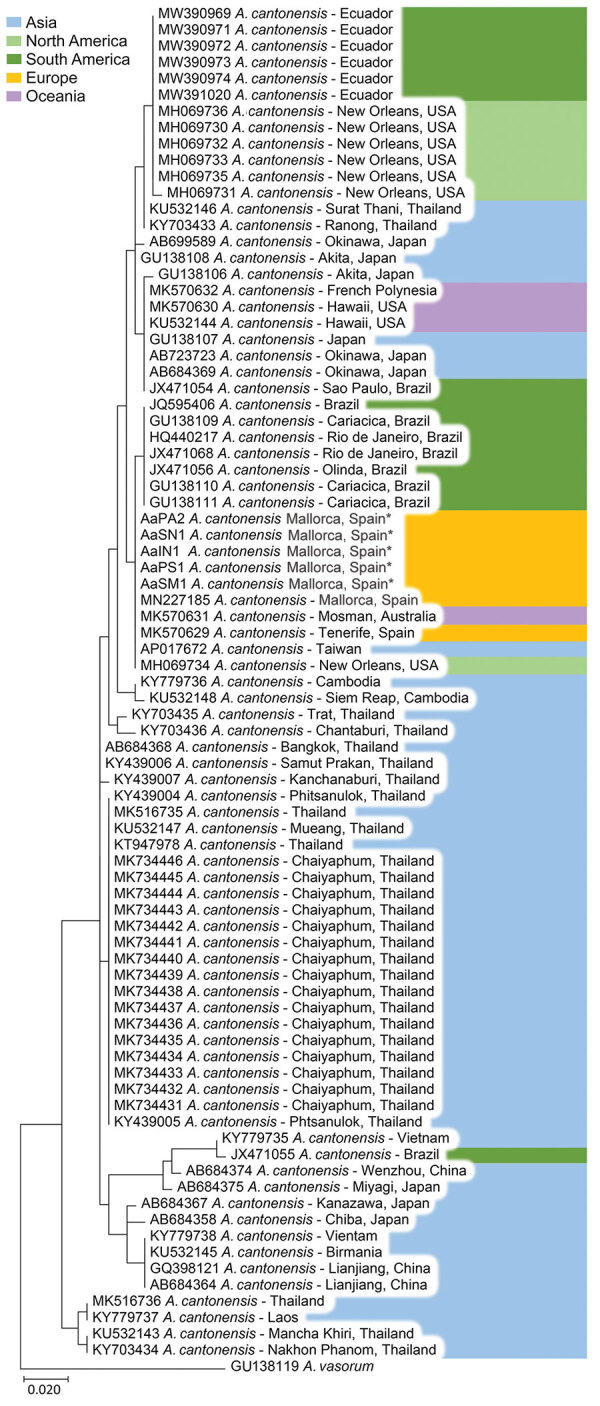
Maximum-likelihood tree showing the phylogenetic position of *Angiostrongylus cantonensis* rat lungworm cytochrome c oxidase subunit I gene fragments generated in study of infected hedgehogs in Mallorca, Spain, 2018–2020 (asterisks), and reference sequences retrieved from GenBank (accession numbers shown).

After ClustalW alignment (https://www.ebi.ac.uk), we obtained a 389-bp sequence of the COI gene region. DNA extraction was not successful for parasites from hedgehog AaAL1. All remaining DNA sequences resulted in the same CI haplotype, the same one that was reported by our group in 2019 (*12*). We subjected the haplotype sequence to BLAST analysis against the GenBank database. The top 78 hits corresponded with COI sequences of *A. cantonensis* nematodes; the first 5 sequences showed 100% identity. Maximum-likelihood analysis resulted in a phylogenetic tree lacking strong bootstrap support values at deeper nodes (Figure 2). Specimens from Mallorca were clustered in the same clade as those from Tenerife (Canary Islands, Spain), Australia, Taiwan, and New Orleans, Louisiana, USA.

## Discussion

This study showed that the invasive neurotropic parasite *A. cantonensis*, the rat lungworm, is the main cause of neurologic disease in North African hedgehogs on the Mediterranean island of Mallorca. The rat lungworm has been found in hedgehogs from 8 locations in Mallorca over 3 consecutive years, indicating that this parasite is spreading and has successfully established in this territory of Europe since 2018.

Sentinel surveillance of hedgehogs that had signs of disease has resulted in a powerful and inexpensive public health monitoring tool to follow invasion of *A. cantonensis* lungworms in Mallorca. Hedgehogs are ubiquitous in Europe, and they have been reported as the most common mammal admitted to wildlife hospitals in Europe, where their clinical signs can be monitored closely (*21*,*22*). Despite the proven utility of this strategy, sentinel surveillance is often underused for detecting emerging pathogens (*23*).

Other mammals have been proposed as sentinels for early detection and understanding of the dynamics of *A. cantonensis* transmission: for example, the tawny frogmouth *Podargus strigoides* in Australia (*24*) because of its abundance and ubiquity (*25*), and dogs because of their clear clinical manifestations (*26*). We found a high (100%) prevalence of *A. cantonensis* worms in animals showing neurologic signs. In positive hedgehogs, the most predictive signs were astasia, lateral recumbency, and bicycling movement. These clinical manifestations might be used for presumptive diagnosis of an *A. cantonensis* infection in wildlife hospitals in Europe. More studies are necessary to validate these observations.

Characteristic neurologic signs of *A. cantonensis* infection have also been observed in tawny frogmouths. Ma et al. detected the parasite in 80% of symptomatic birds, in which paresis/paralysis affecting the hind limbs was the most common clinical manifestation (*25*). Progressive ascending paralysis of the limbs has also been observed in dogs (*27*). The gastropod-borne nematode *C. striatum* was present in most rat lungworm‒positive hedgehogs in our study. This finding is not surprising because the prevalence of this lungworm in Mallorca is high (S. Delgado-Serra, unpub. data) but indicates that both parasites can co-infect the lungs of these mammals. Conversely, eosinophil count was unremarkable. The absence of eosinophilia in peripheral blood has also been observed in other animals (*28*) and humans (*29*) positive for this infection.

We found preliminary evidence of an apparent seasonality of neural angiostrongyliasis in Mallorca; all cases were detected in autumn and early winter (October‒December). This seasonal pattern has also been observed in dogs in eastern Australia (*26*). However, cases in tawny frogmouths, also in eastern Australia, occur in late summer and autumn (*25*). Instead of seasonality, prevalence of neural angiostrongyliasis might reflect periods of increased precipitation because this increase has a direct effect on the availability of snails and slugs (*30*).

Mallorca is an endemic foci of the rat lungworm in Europe; however, intermediate hosts in this region remain to be determined. To date, Egypt and Mallorca are the only rat lungworm–endemic territories in the Mediterranean Basin. Although none of the intermediate hosts reported in Egypt are present in Mallorca, the snail species *Theba pisana* and *Cornu aspersum*, reported in the Canary Islands (*31*) are also present in Mallorca. Both species are widely distributed in continental Europe.

All lungworms we sequenced had the same haplotype and were 100% congruent with those reported in Australia, New Orleans, Taiwan, and Tenerife. The single haplotype found in all specimens might be explained by recent range expansion of this parasite and might be the result of a single colonization event. However, more studies are needed to investigate the invasion origin of this parasite species.

Some open questions and limitations of this study should be discussed. First, we cannot know the exact locations where the parasite is circulating in Mallorca because the extent of the home range of North African hedgehogs can be >90 hectares/day (*32*). Surveillance should then include rats, which have smaller home ranges (*33*), or gastropods, especially because these hosts are far more abundant and widespread than hedgehogs. Second, the data presented do not reflect the real status of neural angiostrongyliasis in hedgehogs in Mallorca because we have only examined animals rescued by citizens. Third, the role of hedgehogs within the living cycle of the parasite is unknown. In 2018, our group found a gravid *A. cantonensis* female worm in the brain of a hedgehog (*12*), indicating that the parasite might reach sexual maturity in this host. However, we found no gravid female subsequently. Whether hedgehogs act as definitive hosts requires further research.

The heavy traffic of ships between the Balearic Islands and continental Europe might have already resulted in the introduction of infected rats to the mainland, and *A. cantonensis* lungworms might be more widely distributed on the continent than previously believed (*5*). Furthermore, the Mediterranean region confronts its own challenges in relation to the arrival of the rat lungworm. Snails are a major part of the Mediterranean diet, which has resulted in an increase of snail farms in the region (*34*). Food safety agencies on the continent might be aware of the increasing challenges for this industry because of possible introduction of the rat lungworm. The first detections of these worms in nonendemic areas often occur after the report of fatal human cases (*35**–**37*). In other regions, infections in wild, domestic, and captive animals have preceded those in humans (*28*), providing the ideal sequence of events to raise early public awareness and to establish early prevention strategies. A delay in the diagnosis and treatment for patients often results in worse prognosis. We recommend adopting a sentinel surveillance and One Health approaches similar to the one we provide in this study for the early detection of the rat lungworm in wildlife hospitals across Europe. However, this strategy should not replace the traditional means of detecting the rat lungworm. Further efforts should include increasing public and medical awareness of neuroangiostrongyliasis and conducting systematic surveillance of rats and gastropods (*38*) in areas across the continent where the rat lungworm is already established or could potentially become established.
