# Supplementary figures and images for: Angiostrongylus cantonensis Nematode Invasion Pathway, Mallorca, Spain
Source: Emerg Infect Dis. 2022 Jun;28(6):1163–9. doi: 10.3201/eid2806.212344 (PMC9155863; doi:10.3201/eid2806.212344)

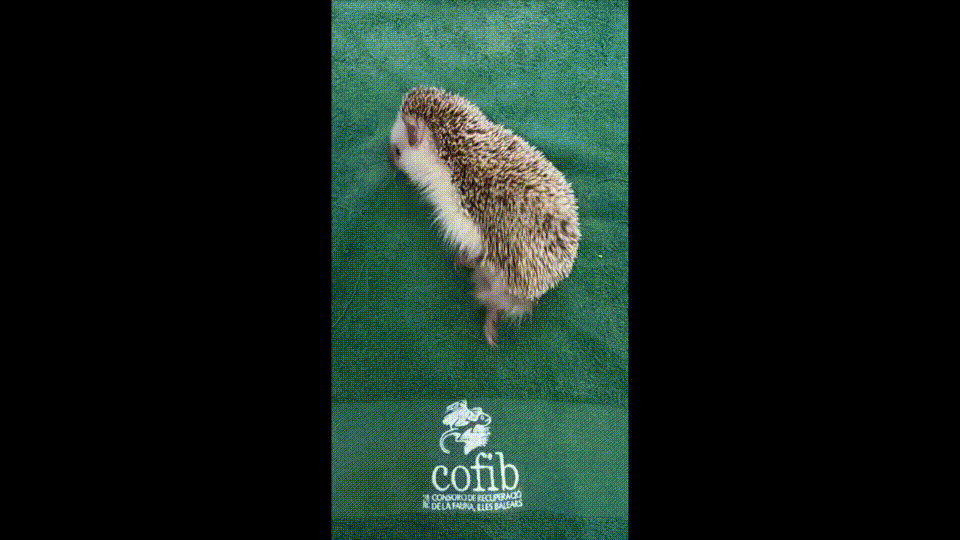

Supplement: Supplementary file 1 [file 21-2344-V.gif]
